# Motion Estimation Using the Firefly Algorithm in Ultrasonic Image Sequence of Soft Tissue

**DOI:** 10.1155/2015/343217

**Published:** 2015-03-19

**Authors:** Chih-Feng Chao, Ming-Huwi Horng, Yu-Chan Chen

**Affiliations:** Department of Computer Science and Information Engineering, National Pingtung University, No. 4-18, Minsheng Road, Pingtung 90003, Taiwan

## Abstract

Ultrasonic image sequence of the soft tissue is widely used in disease diagnosis; however, the speckle noises usually influenced the image quality. These images usually have a low signal-to-noise ratio presentation. The phenomenon gives rise to traditional motion estimation algorithms that are not suitable to measure the motion vectors. In this paper, a new motion estimation algorithm is developed for assessing the velocity field of soft tissue in a sequence of ultrasonic B-mode images. The proposed iterative firefly algorithm (IFA) searches for few candidate points to obtain the optimal motion vector, and then compares it to the traditional iterative full search algorithm (IFSA) via a series of experiments of *in vivo* ultrasonic image sequences. The experimental results show that the IFA can assess the vector with better efficiency and almost equal estimation quality compared to the traditional IFSA method.

## 1. Introduction

The motion estimation not only is an important component of the block-based video compression but also is often applied in disease diagnosis for the image sequences of the ultrasound or the magnetic resonance images. The ultrasonic examination is widely used to present the soft tissues such as heart or muscle; however it is usually influenced by speckle noises and the temporal decorrelation of the speckle patterns [1]. The popular optical flow algorithm [2] is based on the assumption that the intensity of material point of the tissue in a complete image sequence is always invariant. This assumption is generally called intensity invariant constraint; however, there exist the speckle noises and the tissue deformations usually destroy the intensity constraints and therefore cause the difficulty of motion estimation.

Another approach for motion estimation problem is the block-matching algorithm [3–5]; it measures the motion vector by evaluating the similarity criterion, such as the mean square error (MSE), mean absolute error (MAE), and sum of absolute differences (SAD) between the corresponding blocks of two consecutive images. The block-matching algorithm is to find a candidate block within a search region of the corresponding image that best matches the current block in the current image. The displacement between these two matching blocks is called a motion vector (MV). The full search algorithm (FSA) [6, 7] is the simplest block-matching algorithm that can search in burst-force for the optimal estimation solution with minimal matching error as it checks all candidate search points at a time. However, it is extremely computational expensive at the searching process, and, therefore, its usage is seriously limited in the medical image sequences. The effective reduction of the number of search points can accelerate the motion estimation, which is based on the assumption that block distortion is monotonically decreasing. These algorithms are proposed to alleviate the high computational complexity of full search, including the three- or four-step search [8, 9] and the gradient descent search [10]. However, the ultrasonic images are usually noisy and with low signal-to-noise ratio due to the speckle noises and the decorrelation of the speckle patterns; as a result the monotonically decreasing assumption does not hold such that the usage of these algorithms always traps into local optima. In order to tackle this problem, the traditional motion estimation algorithms are revised into an iterative algorithm together with a specific smoothness constraint; however, these changes need a great amount of computation to estimate optimal motion vectors when we use the iterative full search algorithm (IFSA).

Recently, the algorithms of bioinspired computing (BIO) such as the honey bee mating algorithm [11] and shuffled frog-leaping algorithm [12] have been used to efficiently search for the global optimum in the complex optimization problems. Up to the present, there are few of BIO methods that addressed the problems of motion estimation in the ultrasonic image sequence. Among them, the firefly algorithm is another algorithm that had been used in the image thresholding and vector quantitation [13, 14]. In this paper, we present an iterative algorithm that builds in the firefly algorithm for motion estimation. The proposed algorithm is introduced in Section 2. In Section 3, we demonstrate the experimental results. The conclusion and final remarks are given in Section 4.

## 2. Material and Methods

The problem of estimating motion field from image sequence has been studied extensively. The block-matching algorithm is the most popular because of its simplicity.  Section 2.1 will briefly describe the block-matching algorithm.  Section 2.2 introduces the firefly algorithm. In order to handle the problem of speckle noises, we designed an iterative firefly algorithm (IFA) together with a smooth constraint to estimate the motor vectors, described in Section 2.3.

### 2.1. The Block-Matching Algorithm

**Figure 1:**
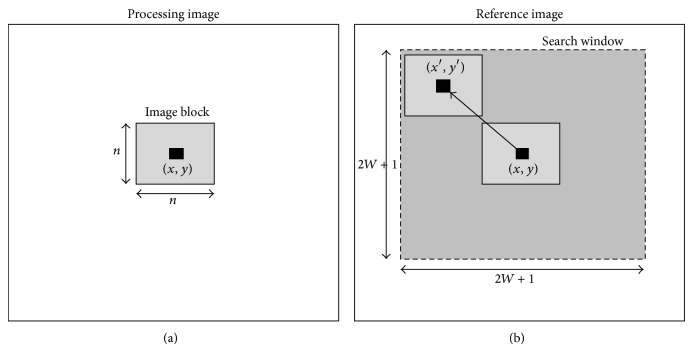
Illustration of the block-matching algorithm [18].

Because of its simplicity, the block-matching is a widely used algorithm in motion estimation. In matching procedures, the estimated image block of the processing frame will correspond to the best matching location within the predefined search window of the reference frame, as shown in Figure 1. In general, the size of estimated block,  **B** is  *n* × *n*  pixels and the size of the corresponding search window **S** is (2*W* + 1) × (2*W* + 1). These two windows are centered at the same point  (*x*, *y*)  in the two consecutive image frames (*f*_*k*−1_  and  *f*_*k*_). The full search searches for all possible locations within the search window by evaluating some matching criteria and selected one location  (*x*′, *y*′)  of the corresponding block  **B** ^*^. The relative displacement of the two locations of both  (*x*, *y*)  and (*x*′, *y*′) is defined as the motion vector,  *u*_*B*_ = (*u*_*Bx*_, *u*_*By*_),  *u*_*Bx*_ = *x*′ − *x*, *u*_*By*_ = *y*′ − *y*.

The estimation of the motion vector is to find the optimal one such that its corresponding mean square error (MSE) is the smallest. Equation (1) defines the MSE measure:

(1) MSEuBx,uBy =1N×N∑i=1N ∑j=1Nfkm,n−fk−1m+uBx,n+uBy2,             −W≤uBx, uBy≤W.

In other words the optimal motion vector  *u*_B_^*^ = (*u*_Bx_^*^, *u*_By_^*^) satisfies the following equation:

(2) $$\begin{array}{c} u_{B}^{*}=\text{Arg}\;\text{MIN}\left\{\text{MSE}\left(u_{Bx},u_{By}\right)\right\}. \end{array}$$

### 2.2. Firefly Algorithm

The firefly algorithm is a new bioinspired computing approach for stochastic optimization, in which the search mechanism is simulated by social behavior of fireflies and the phenomenon of bioluminescent communication. There are two important issues in the firefly algorithm that are the variation of light intensity and formulation of attractiveness. Yang [15] simplifies that the attractiveness of a firefly is determined by its brightness which in turn is associated with the specific fitness function. The attractiveness is proportional to their brightness. Every member *x*_*i*_ of the firefly swarm is characterized by its bright *I*_*i*_ in which one can directly measure the corresponding fitness value.

Furthermore, there are three idealized rules.  (1)  Regardless of their sex, any one firefly will be attracted to other fireflies.  (2)  Attractiveness is proportional to their brightness, so for any two flashing fireflies, the less bright one will move to the brighter one. If there is brighter one than the particular firefly, it will move randomly.  (3)  Brightness of a firefly is affected or determined by the landscape of the given fitness function  *φ*(*x*); in other words, the brightness  *I*(*x*_*i*_)  of a firefly  *x*_*i*_ can be defined as *φ*(*x*_*i*_).

More precisely, the attractiveness between fireflies  *x*_*i*_  and  *x*_*j*_  is defined as any monotonically decreasing function of their distance  *r*_*ij*_ by the following equations:

(3) $$\begin{array}{c} r_{i,j}=\left\|x_{i}-x_{j}\right\|=\sqrt{\overset{c}{\underset{k=1}{\sum }}{\left(x_{i,k}-x_{j,k}\right)}^{2}},\\ \beta ⟵\beta_{0}e^{-{\gamma r}_{i,j}}, \end{array}$$

where  *β*_0_  is the sum of initial assigned brightness of these two fireflies, *γ* is the light absorption coefficient, and  *k*  indicates the index of the dimension of the candidate solution (i.e., firefly).

The movement of a firefly  *x*_*i*_  that is attracted to another more attractive firefly  *x*_*j*_  is determined by the following equations:

(4) xi,k⟵1−βxi,k+βxj,k+ui,k,ui,k=rand  1−12.

If there is no brighter one than a particular firefly,  *x*_*i*^max⁡^_, it will move randomly according to the following equation:

(5) ximax⁡,k⟵ximax⁡,k+uimax⁡,k,uimax⁡,k=rand  2−12,

where rand1  and  rand2  are random numbers obtained from the uniform distribution  *U*(0, 1).

### 2.3. The Proposed Motion Estimation Algorithm Using the Firefly Algorithm

The firefly algorithm usually defines a fitness function to compute the brightness of fireflies. Traditionally, the motion estimation algorithms use a similarity measure as their fitness function such as the mean square error (MSE) [3, 4]. However, the fact that speckle noises are exhibited in ultrasonic image sequence so that the traditional similarity criterion is not enough to accurately estimate motion vectors. To further decrease the sensitivity to speckle noises, a smoothness constraint is adopted to force continuity among neighbors motion vectors. Therefore, the used fitness function of the proposed algorithm consists of two terms of the similarity measure and the smoothness constraint. The widely used similarity measure is the mean square error (MSE), when the best motion vector is found; its corresponding MSE is the minima. The smoothness constraint is necessary to refer to its neighbors pixel's motion vector to estimate current motion vector, and therefore it must be iterative. More practically, the calculation of motion vector at iterative number *t* must consider the motion vectors of its neighbors at iterative number  *t* − 1. The detail of the designed smoothness constraint is described as follows.

Suppose that the estimating motion vector of current block  **B**  with center pixel  **P**  at iterative number *t* be the *u*_*B*_^*t*^ = (*u*_*Bx*_^*t*^, *u*_*By*_^*t*^), and then the estimated motion vectors of block $\bar{B_{i}}$ with the center of pixel $\bar{P_{i}}$ at iterative number *t* − 1 are $u_{{\overset{-}{B}}_{i}}^{t-1}=(u_{\bar{B_{i}}x}^{t-1},u_{\bar{B_{i}}y}^{t-1})$. The $\bar{P_{i}}\;\;(i=1,2,\ldots ,8)$ is the eight neighbors of point *P*. The smooth constraints (SC) at the iteration *t* are described in the following equation:

(6) SCuBt=SCuBxt,uByt=18∑i=18uBxt−uBi¯xt−12+uBxt−uBi¯xt−12.

The fitness function is defined as (7); it is used as search criteria for estimating motion vector in this paper:

(7) Fittness  uBt=11+MSEuBxt,uByt+λSCuBxt,uByt,      the  λ  is  a  smooth  parameter.

The proposed algorithm is described as follows.

**Step 1 (setup the parameters of proposed system).**

This step assigns the parameter of the number fireflies  (*m*), the maximum iteration number  (*l*)  and the light absorption coefficient  (*γ*), and the smooth parameter (*λ*).  In addition, according to (6), the estimation of the motion vector of block *B* needs these initial motion vectors of neighbor$\;\bar{B_{i}}$. Therefore, all of the initial motion vectors of pixels of estimating block are randomly given ranging from −*W* to *W*.

**Step 2 (assign the initials of each firefly).**

For each estimating block *B*, we assigned *m* fireflies (in general, *m* ≪ (2*W* + 1) × (2*W* + 1)) and each firefly is assigned an initial motion vector *u*_*iB*_^0^ ranging from −*W* and *W*; that is,

(8) $$\begin{array}{c} u_{iB}^{0}=\left(u_{iBx}^{0},u_{iBy}^{0}\right),\;-W\leq u_{iBx}^{0},\;\;u_{iBy}^{0}\leq W. \end{array}$$

  
*t*  denotes the iterative number and is initially assigned to be 0 and the initial brightness of each firefly is assigned by its corresponding fitness.

**Step 3 (update all candidate solutions).**

The mechanism of updating of candidate solution is generally stochastic; more practically, the IFA sequential picked up current *i*th firefly solution and randomly selects its corresponding *j*th firefly solution. The two motion vectors are denoted by *u*_*iB*_^*t*^ = (*u*_*iBx*_^*t*^, *u*_*iBy*_^*t*^) and *u*_*jB*_^*t*^ = (*u*_*jBx*_^*t*^, *u*_*jBy*_^*t*^).  If the fitness value of *u*_*iB*_^*t*^ is less than the fitness of *u*_*jB*_^*t*^, *u*_*iB*_^*t*^ will update toward *u*_*jB*_^*t*^ according to the following equations:

(9) ri,j=uiBt−ujBt=uiBxt−ujBxt2+uiByt−ujByt2,β⟵β0e−γri,j,w=wx,wy  is  a  random  walk  ranged  with−0.5<wx, wy<0.5,

where *β*_0_ is the sum of initial assigned brightness (fitness value) of these two fireflies and *γ* is the light absorption coefficient:

(10) uiBxt,uiByt =1−βuiBxt,uiByt+βujBxt,ujByt+wx,wy.

All of the candidate solutions are updated according to the procedures of (9) and (10), and then the best one is calculated for later process.

If the  *i*th firefly is the best one among all fireflies, this firefly will perform a random walk to renew its solution by using (11). If the new one has better fitness than the original one, it will replace it with the best solution; otherwise it is discarded. Finally, the best solution will be recorded by  *u*_BestCu_:

(11) uiBxt,uiByt=uiBxt,uiByt+wx,wy,w=wx,wy  is  a  random  walk  ranged  with−0.5<wx, wy<0.5.

**Step 4 (iterative execution and resulting vector output).**

The iteration number *t* adds by 1: If  *t*  reaches the maximum iteration number *l*, the algorithm outputs the *u*_BestCu_ as the resulting motion vector; else go to Step 3.

## 3. Experimental Results

### 3.1. Execution Environment

All programs are programed by using Visual C++ programming language on a personal computer 2.4 GHz CPU, 1 G RAM running window XP system. All experiments were conducted in parameters of light absorption coefficient,  *γ* = 1.0. The size of initial firefly is assigned to be 5 and the maximum iteration number  *l* = 100. The sizes of estimated image region, estimated block, and search window are  25 × 25, 7 × 7, and 15 × 15  (i.e., *W* = 7).

**Figure 2:**
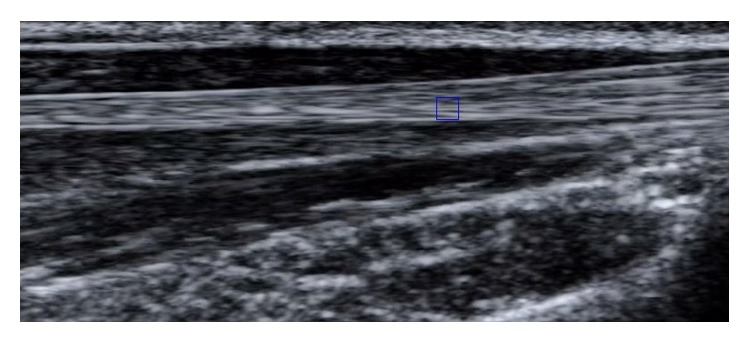
An image of ultrasonic extensor tendon image sequence.

Because of the substantial computational power of modern ultrasound systems, it is potential to evaluate muscle motion in real time [1]. The forearm extensors and extensor tendon are at the back of the forearm that typically cause pain at the outer aspect of the elbow. These muscles and tendon act to extend the wrist and fingers to bend the wrist backwards and straighten the fingers. In experiments, images were obtained by scanning the extensor tendon in longitudinal cross section. The images were taken from the tendon portions when the subject's wrist moved extension to flexion.  Figure 2 shows an ultrasonic extensor tendon image sequence, whose frame rate is 39 frames of one second.

### 3.2. The Selection of Smooth Parameter *λ*

The setting of smoothness parameter may influence the estimation quality of motion estimation algorithm; we explored the different smoothness parameters in experiments. In general, the estimation quality is characterized by the peak signal-to-noise (PSNR) value; more practically, in PSNR the signal is the original data frames; meanwhile, the noise is the error introduced by the calculated motion vectors. The definition of PSNR is expressed as

(12) $$\begin{array}{c} \text{PSNR}=10\times \log_{10}\left(\frac{255^{2}}{\text{MSE}}\right). \end{array}$$

**Figure 3:**
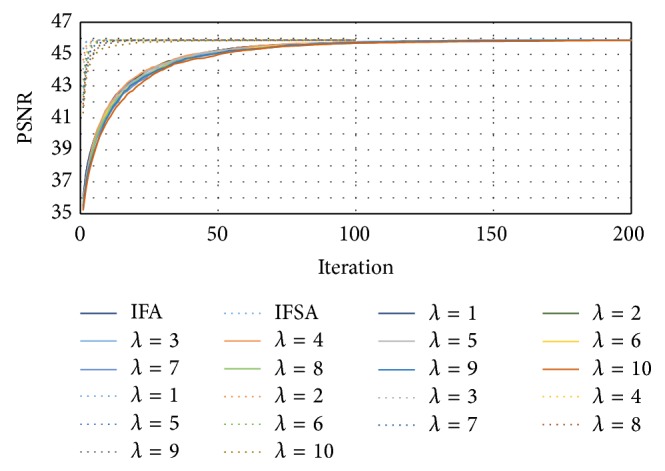
The corresponding PSNR and the iteration number of different smoothness parameters.

The MSE is the mean square error between the original frames and those compensated by the estimated motion vectors.  Figure 3 showed an illustration of results of PSNR versus the iteration number under different smoothness parameters. Form this figure, we found that the setting of the smoothness parameter was not significant to the final results of IFA and IFSA algorithms, and further the IFSA is in need of 20 iterations to converge but the iterative firefly algorithm requires 200 iterations. As a result, the smoothness parameter of all follow-up experiments was assigned to be 1.0.

### 3.3. Performance Evaluation

For comparisons with two different algorithms, two relevant performance indices were considered: the estimation quality and the search efficiency.

First the IFSA and IFA algorithms were compared in terms of estimation quality. The widely used index is the PSNR defined as (12); in addition to PSNR, an alternative index called PSNR degradation ratio (*D*_PSNR_) [5] is used in the experiments, which expresses the level of mismatch between the PSNRs of IFSA and IFA methods. The *D*_PSNR_ is defined as

(13) $$\begin{array}{c} D_{\text{PSNR}}=\left(\frac{{\text{PSNR}}_{\text{IFSA}}-{\text{PSNR}}_{\text{IFA}}}{{\text{PSNR}}_{\text{IFSA}}}\right)\times 100\%. \end{array}$$

**Figure 4:**
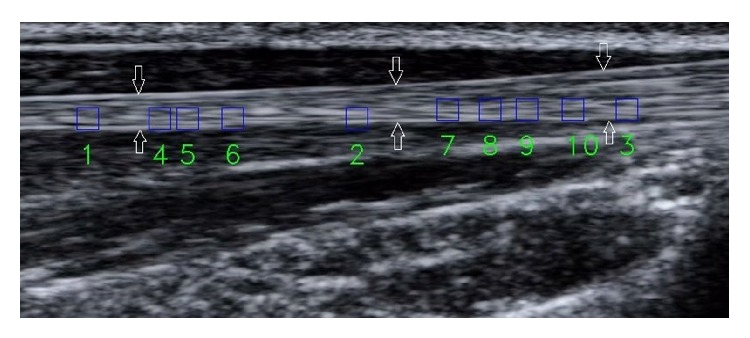
The selected 10 regions of interest of the extensor tendon portion indicated by arrows.

**Figure 5:**
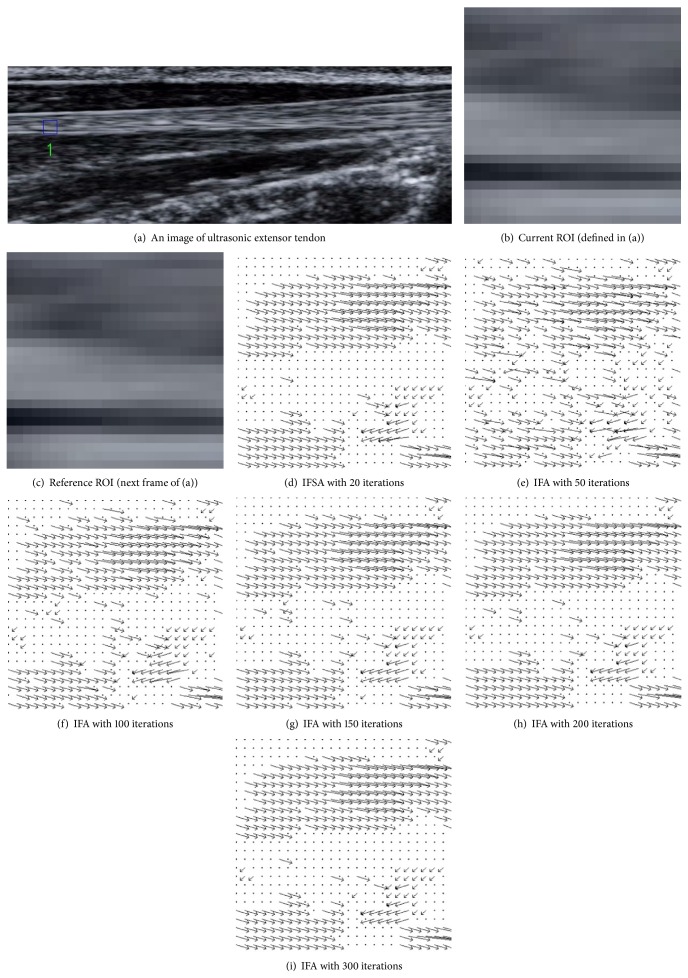
The estimated motion vectors by using the IFSA and IFA with different iterations.

Figure 4 shows the 10 selected regions of interest (ROI) with size of  25∗25  pixels in an ultrasonic extensor tendon image sequence; those are used to estimate their motion vector by using the IFA and IFSA. Tables 1 and 2 show the PSNR values and computational times under running 200 iterations, considering the ten ROIs of Figure 4. Tables 3 and 4 are the results of PSNR versus computational time under 300 iterations. As it can be seen from these tables the quality results of both the IFA and IFSA have a limited difference. The corresponding PSNR degradation ratios are only 0.02105% (200 iterations) and 0.002% (300 iterations). The little differences imply that the usage of IFA is an alterative to calculate the accurate motion vectors.  Figure 5 shows the resulting motion vectors of the first ROI using the IFSA and IFA ran with different iterations. In this figure, the interval time between current ROI and reference ROI is 1/39 second.

In addition, the computational times of the IFA and IFSA methods serve as an index for measuring the search efficiency. The index call computation saving ratio (CSR) [5] is defined for comparison in the experiments. It expresses the level of computation saving ratio between the computational times of the IFA and IFSA. The CSR is defined as

(14) $$\begin{array}{c} \text{CSR}=\left(\frac{{\text{Time}}_{\text{IFSA}}-{\text{Time}}_{\text{IFA}}}{{\text{Time}}_{\text{IFSA}}}\right)\times 100\%. \end{array}$$

Tables 1 and 3 show the computational time of estimating of the 10 ROIs using both IFSA and IFA. From the two tables we found that the average computational time of IFA is 8.20 seconds (200 iterations) and 12.301 seconds (300 iterations), whereas the IFSA needs 30.785 seconds (20 iterations). Tables 5 and 6 show that the average CSRs of IFA are 73.4163% (200 iterations) and 60.1631% (300 iterations); in other words, IFA can save in excess of 60% computational cost.

## 4. Conclusion

Based on the results of the present experiments of ultrasonic extensor tendon image sequence, the following conclusions can be emphasized.

1. In spite of the fact that the IFSA is a widely used algorithm to calculate the motion vector, it needs a great amount of computational time to calculate motion vectors, especially in the medical image sequences.
2. The IFA method is a stochastic algorithm that searches for optimal solution according to the swarm behavior of fireflies. This mechanism can effectively tackle the influences of speckle noises.
3. Experimental results revealed that the estimation qualities of the IFA and IFSA are nearly equal, and the IFA can save in excess of 60% computational cost. It implies that the proposed IFA is a more powerful algorithm in motion estimation of the application of ultrasonic image sequence.
4. The IFA pioneered in an application of bioinspired algorithm in estimating the motion field of ultrasonic image sequence. Maybe we can further investigate other bioinspired algorithms in the same application motion estimation. In addition, the IFA is a potential to be extensively applied in other motion researches such as motion vector composition [16, 17].

## Figures and Tables

**Table 1:** The PSNR and computational time of both IFSA and IFA which runs 200 iterations.

Extensor tendon	IFSA with 20 iterations			IFA with 200 iterations		
	MSE	PSNR (dB)	Time (sec)	MSE	PSNR (dB)	Time (sec)
ROI 1	2.685	43.842	31.205	2.687	43.838	8.323
ROI 2	1.800	45.578	30.549	1.802	45.573	8.167
ROI 3	1.924	45.289	30.740	1.930	45.274	8.219
ROI 4	2.047	45.020	30.455	2.052	45.008	8.157
ROI 5	2.525	44.109	31.095	2.530	44.100	8.251
ROI 6	2.693	43.828	30.690	2.699	43.818	8.198
ROI 7	1.676	45.889	30.424	1.681	45.876	8.115
ROI 8	2.020	45.078	30.439	2.027	45.063	8.115
ROI 9	1.741	45.723	30.937	1.743	45.718	8.230
ROI 10	1.718	45.781	31.315	1.725	45.764	8.229

Average	2.083	45.014	30.785	2.088	45.003	8.200

**Table 2:** The PSNR and *D*_PSNR_ of the usages of both IFA and IFSA, as it can be seen that the average of *D*_PSNR_ is only 0.02105% when IFA runs 200 iterations.

PSNR	ROI 1	ROI 2	ROI 3	ROI 4	ROI 5	ROI 6	ROI 7	ROI 8	ROI 9	ROI 10
IFSA	43.842	45.578	45.289	45.020	44.109	43.828	45.889	45.078	45.723	45.781
IFA	43.838	45.573	45.274	45.008	44.100	43.818	45.876	45.063	45.718	45.764
*D*_PSNR_ (%)	0.0091	0.0109	0.0110	0.0266	0.0204	0.0228	0.0283	0.0332	0.0111	0.0371

Average of *D*_PSNR_ (%)						0.02105				

**Table 3:** The PSNR and computational time of both IFSA and IFA after running 300 iterations.

Extensor tendon	IFSA with 20 iterations			IFA with 300 iterations		
	MSE	PSNR (dB)	Time (sec)	MSE	PSNR (dB)	Time (sec)
ROI 1	2.685	43.842	31.205	2.685	43.842	12.485
ROI 2	1.800	45.578	30.549	1.800	45.578	12.250
ROI 3	1.924	45.289	30.740	1.926	45.284	12.329
ROI 4	2.047	45.020	30.455	2.047	45.021	12.235
ROI 5	2.525	44.109	31.095	2.525	44.109	12.376
ROI 6	2.693	43.828	30.690	2.693	43.828	12.297
ROI 7	1.676	45.889	30.424	1.677	45.887	12.173
ROI 8	2.020	45.078	30.439	2.022	45.073	12.173
ROI 9	1.741	45.723	30.937	1.741	45.723	12.345
ROI 10	1.718	45.781	31.315	1.718	45.783	12.344

Average	2.083	45.014	30.785	2.083	45.013	12.301

**Table 4:** The PSNR and *D*_PSNR_ of the usages of both IFA and IFSA, as it can be seen that the average of *D*_PSNR_ is only 0.002% after running 300 iterations.

PSNR	ROI 1	ROI 2	ROI 3	ROI 4	ROI 5	ROI 6	ROI 7	ROI 8	ROI 9	ROI 10
IFSA	43.842	45.578	45.289	45.020	44.109	43.828	45.889	45.078	45.723	45.781
IFA	43.842	45.578	45.284	45.021	44.109	43.828	45.887	45.073	45.723	45.783
*D*_PSNR_ (%)	0.0000	0.0000	0.0110	−0.0022	0.0000	0.0000	0.0044	0.0111	0.0000	−0.0043

Average of *D*_PSNR_ (%)						0.002				

**Table 5:** The computational time and the CSR of the usages of both IFA and IFSA, as it can be seen that the average of CSR is 73.416% under 200 iterations.

Time (sec)	ROI 1	ROI 2	ROI 3	ROI 4	ROI 5	ROI 6	ROI 7	ROI 8	ROI 9	ROI 10
IFSA	31.205	30.549	30.740	30.455	31.095	30.690	30.424	30.439	30.937	32.315
IFA	8.323	8.167	8.219	8.157	8.251	8.198	8.115	8.115	8.230	8.229
CSR (%)	73.327	73.266	73.556	73.465	73.465	73.287	73.329	73.340	73.398	73.722

Average of CSR (%)						73.4163				

**Table 6:** The computational time and the CSR of the usages of both IFA and IFSA, as it can be seen that the average of CSR is 60.1631% under 300 iterations.

Time (sec)	ROI 1	ROI 2	ROI 3	ROI 4	ROI 5	ROI 6	ROI 7	ROI 8	ROI 9	ROI 10
IFSA	31.205	30.549	30.740	30.455	31.095	30.690	30.424	30.439	30.937	32.315
IFA	12.485	12.250	12.329	12.235	12.376	12.297	12.173	12.173	12.345	12.344
CSR (%)	59.990	59.900	59.892	59.825	60.199	59.931	59.989	60.008	60.096	61.801

Average of CSR (%)						60.1631				
